# Decline in attentional inhibition among migraine patients: an event‐related potential study using the Stroop task

**DOI:** 10.1186/s10194-021-01242-6

**Published:** 2021-05-03

**Authors:** Min Su, Rongfei Wang, Zhao Dong, Dengfa Zhao, Shengyuan Yu

**Affiliations:** Department of Neurology, the First Medical Center, Chinese PLA General Hospital, 100853 Beijing, China

**Keywords:** Migraine, Attentional inhibition, Event‐related potentials, Stroop effect, Allodynia

## Abstract

**Background:**

As a disorder of brain dysfunction, migraine has been associated with cognitive decline. However, no consistent results with respect to the attention function in migraineurs have been found, and the relationship between attentional inhibition and migraine is also unclear. In this study, the attentional inhibition function was evaluated using event-related potentials (ERPs) while migraine patients and healthy controls were performing the color–word Stroop task.

**Methods:**

In this study, 75 migraine patients and 41 age-, gender-, and education-matched healthy controls were enrolled. The Stroop task was performed, and both behavioral and ERP data were analyzed.

**Results:**

As to the behavioral data, the migraine group had a longer reaction time compared to the control group, but no difference in Stroop effect was observed. With respect to ERP components, the amplitudes of both early and late medial frontal negativity (MFN) were decreased in the migraine group. Additionally, obvious differences in the early MFN and sustained potential (SP) amplitudes were found between patients with and without allodynia.

**Conclusions:**

At the behavioral level, migraine patients exhibited decreased executive ability but no obvious decline in inhibition. By contrast, a decline in attentional inhibition during the migraine interictal phase was confirmed by the analysis of ERP components, mainly those associated with changes in the conflict-monitoring stage, independent of confounding factors such as age, education, medication and mood disorders. Migraine patients with allodynia exhibited some significant differences in early MFN and SP compared to those without, supporting the hypothesis that migraine chronification aggravates the decline in attentional inhibition.

## Introduction

Migraine is a disease entity that includes headache and concomitant autonomic symptoms, hypersensitivity to other stimuli, mood responses, and cognitive deficits [1]. The cognitive aspect of migraine is not fully understood, but it is notable for its effect in exaggerating disability due to the disease. Migraine is related to some areas of cognitive decline, such as processing speed, memory, planning, attention, concentration, judgment, language, and so on [2]. Clinically, worse pain severity is always associated to more severe cognitive decline; vice versa, more severe decreased cognitive performance is also related to high levels of disability induced by migraine attacks [3, 4]. Until now, the relationship between migraine and cognitive decline has been unclear; as both reflect brain dysfunction, the two may influence each other.

Cognitive disturbances have been reported by migraine patients during all phases of migraine from the premonitory to the postdrome phase, as well as in the interictal phase [1]. And poor cognitive performance during migraine attacks has been generally confirmed in clinical studies, while the interictal data are conflicting [5]. On one hand, migraineurs show impaired cognitive function interictally in part of clinic-based studies, but not in population-based studies; on the other hand, preventive medications and comorbid disorders (mainly including depression, anxiety and sleeping disorder) seem to impact cognitive function in migraine, but cannot fully explain the cognitive decline induced by migraine.

A decline in attention has been widely reported in chronic painful conditions, and the fear–avoidance model of the relationship between pain and attention has attempted to address this [6, 7]. However, the assessment of attention among migraine patients has not yielded consistent results. Recent studies demonstrated that migraineurs exhibited heightened attention to irrelevant visual stimuli [8, 9], but negative results were reported in neuropsychological tests of attention [10, 11]. Hence, in this paper, event-related potentials (ERPs) were chosen to assess attentional functioning in migraine patients with greater sensitivity.

The Stroop task has been widely used for attention assessment, especially in the domain of attentional inhibition [12]. In this task, participants are asked to identify the color of a word stimulus during congruent (the color and meaning of the word are consistent) and incongruent trials (the color and meaning of the word are incompatible). Subjects always need more time to respond in incongruent than in congruent trials, and this increased reaction time is defined as the Stroop effect. During the incongruent trials, individuals need to suppress word information and respond only to the color information. Therefore, a smaller Stroop effect represents better inhibition, and an increase in the Stroop effect results from a decline in the efficiency of inhibitory processes [13].

The Stroop task has been widely used to examine the ERP correlates of cognitive processes related to selective attention, and two main components have been identified to distinguish incongruent trials from congruent trials [14, 15]. First, medial frontal negativity (MFN) or N450, a negative potential extending over the midline frontal region of the scalp between 300 and 500 + ms after stimulus onset, is strongly related to the conflict-detection process rather than to response selection or conflict resolution [16]. MFN can be divided into two subcomponents—the early one is correlated with stimulus conflict detection, and the late one with response conflict detection. Second, the parietal sustained potential (SP), a sustained positivity over the parietal region of the scalp lasting between 500 and 1,000 ms after stimulus onset, is related to the process of conflict resolution [17].

The overall aim was to investigate the electrophysiological correlates of selective attention in migraine using ERPs in response to the Stroop task in migraine patients during the interictal phase, independent of possible confounding factors, such as medication, psychiatric disorders and sleeping disorder. Also, behavioral data including response time were analyzed. With these analyses, we expected to assess whether migraine patients exhibited deficits in attentional inhibition from both behavioral and electrophysiological perspectives.

## Methods

A total of 75 migraine patients (51 migraine without aura, 16 migraine with aura, and 8 chronic migraine patients), identified according to the third version of the International Classification of Headache Disorders [18], were recruited from Chinese PLA General Hospital, and 41 healthy age-matched volunteers with no history of headache were recruited. The study was approved by the Ethics Committee of the Chinese PLA General Hospital in accordance with the ethical principles of the Declaration of Helsinki. Written consent was obtained from all subjects prior to the experiment.

All migraine patients participated during the interictal phase; that is, the patient had no attacks within 48 h before and 24 h after the recording. Participants with any of the following conditions were excluded: other types of headache or chronic pain conditions; regular use of medication, including alcohol and psychotropic drugs; history of mental disease, stroke, brain injury, and/or other neurological disorder; education duration less than 9 years; depression, anxiety, or sleep disorder (score more than 7 points in Hamilton Anxiety/Depression Scale); obvious cognitive dysfunction (score less than 24 points in Mini-Mental State Examination or less than 26 points in Montreal Cognitive Assessment); a history of medication-overuse or prophylactic treatment during 3 months before assessment; consumption of any medication within 3 days before the recording; and uncorrected visual or hearing disability.

Before the Stroop task, the following data were recorded: (1) demographic data including age, gender, educational experience, and body mass index; (2) data associated with headache including subtype of migraine, duration, headache frequency (headache days per month over the previous 3 months), numeric rating scale score of headache, positive family history or not, the presence of allodynia (which was assessed via clinical inquiry according to previously reported possible items such as combing, shaving, wearing glasses or tight cloths, showering and so on [19]), and Headache Impact Test-6 (HIT-6) score; (3) cognitive assessment scores using the Mini-Mental State Examination (MMSE) and Montreal Cognitive Assessment (MoCA) test; and (4) mental assessment scores using the Hamilton Anxiety Scale (HAMA) and Hamilton Depression Scale (HAMD).

For the Stroop task, subjects were asked to identify the color or name of a word by pressing one of four keys (D, F, J, or K, each representing one color) on a computer keyboard using the index and middle fingers of both hands. As described by West [13], the experiment was divided into a color-to-key acquisition phase, a practice phase, and a test phase. During the test phase, four kinds of trials were presented against a black background: congruent (e.g., “RED” printed in red letters), incongruent (e.g., “RED” in blue letters), neutral (e.g., “BIG” in blue), and word identification trials (e.g., “RED” in white letters). In the word identification trials, participants were instructed to press the key identifying the meaning of the word, whereas they responded to the color of the word in other kinds of trials.

**Fig. 1:**
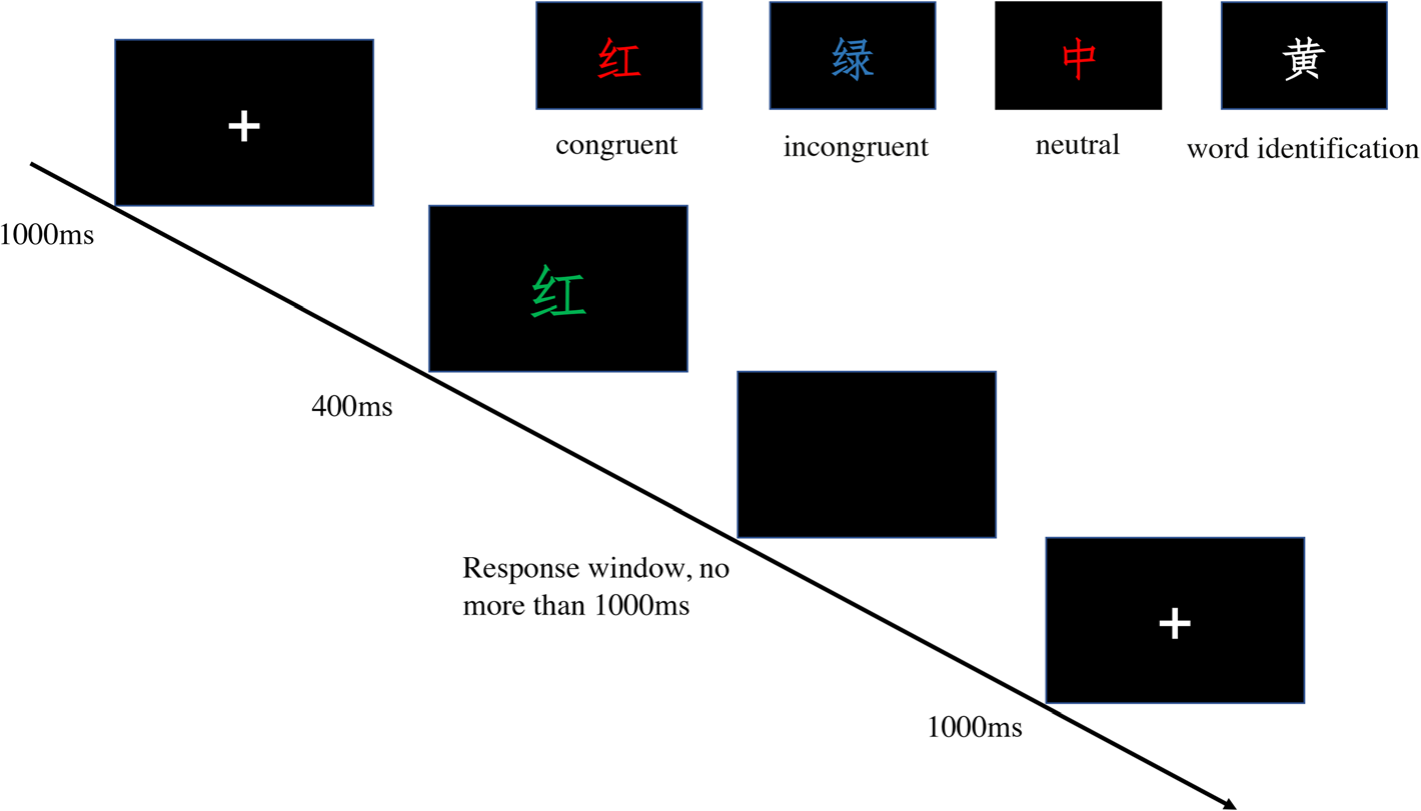
*The Stroop task paradigm.* During the test phase, four kinds of trials were presented randomly against a black background: congruent (e.g., “RED” printed in red letters), incongruent (e.g., “GREEN” in blue letters), neutral (e.g., “ZHONG” in blue), and word identification trials (e.g., “YELLOW” in white letters). In the word identification trials, participants were instructed to press the key identifying the meaning of the word, whereas they responded to the color of the word in other kinds of trials

A total of 384 trials were equally divided into four blocks for the test phase (Fig. 1). In the 96 trials in each block, equal numbers of congruent, incongruent, neutral, and word identification stimuli were randomly presented. At the beginning of each block, a message appeared on the screen instructing the participants to press the space bar to start. After the space bar was pressed, a fixation cross was presented for 1,000 ms, and then a stimulus was presented in the center of the screen for 400 ms, followed by a blank screen for a response (which disappeared after the key was pressed, no more than 1,000 ms). After the response window disappeared, a new trial began. Subjects could rest for 2–4-minutes between blocks. The task was programmed using E-prime software (Psychology Software Tools, USA), and the response time was recorded automatically.

Electroencephalogram (EEG) data (band pass 0.05–100 Hz, sampling rate 500 Hz) were continuously recorded from an array of 64 electrodes referenced to the left mastoid (the right mastoid was the recording site) based on the 10/20 system using Neuroscan Amplifier and Curry Neuroimaging Suite 7.0 software (Compumedics Neuroscan, USA). Vertical and horizontal eye movements (VEOG and HEOG, respectively) were recorded from electrodes placed lateral to and below the right and left eyes. The electrode impedance was maintained below 5 kΩ during the entire experiment.

EEG data were analyzed offline using Curry software. After re-referencing and artifact correction, the ERP epochs were extracted offline, including 200-ms pre-stimulus and 1,500-ms post-stimulus activities. For trials with a correct response, EEG segments were averaged separately for different stimulus types (i.e., congruent and incongruent). The peak amplitudes of two ERP components, MFN and SP, were measured relative to the pre-stimulus baseline period. MFN was defined as a negative peak between 250 and 550 ms and divided into 400-ms early and late MFN epochs. SP was defined as a positive peak between 500 and 1,000 ms.

All data were analyzed using SPSS 19.0 software (IBM Corp., USA). Levene’s test for homogeneity was conducted to test the data distributions. The chi-square test was used to compare qualitative data, and quantitative data were analyzed using analysis of variance (ANOVA) or t-tests. All data are expressed as the mean ± standard deviation, and a *p*-value < 0.05 was considered to indicate significance. Of note, the amplitudes of the MFN and SP components were analyzed using repeated-measures ANOVA, with task type (congruent versus incongruent) and electrode site (F1, FZ, F2, FC1, FCZ, or FC2 for MFN; F8, FT8, F7, FT7, P3, PZ, or P4 for SP) as within-subject factors and group (migraine versus control) as between-subject factors. The degrees of freedom were corrected using the Greenhouse-Geisser epsilon, and simple effect analyses were conducted to explore interaction effects.

## Results

### Demographic data

Seventy-five migraine patients and 41 healthy controls were included; no significant differences in age, gender, or education level were found between the two groups (*p* > 0.05). The migraine group consisted of 51 migraine patients without aura (MO), 16 migraine patients with aura (MA), and eight chronic migraine (CM) patients. The patients’ clinical characteristics are summarized in Table 1. Of notes, 16 patients (2 MA, 2 CM and 12 MO patients) reported the presence of cephalic cutaneous allodynia, and three of them presented as ictal allodynia with the others reported both ictal and interictal allodynia.

**Table 1:** Demographic data

	Migraine(*n*=75)	Control (*n*=41)
Gender(female)	51(68%)	23(56.09%)
Age	31.87±7.32	31.07±5.61
Education		
≤12 years	18	7
>12 years	57	34
BMI(kg/m^2^)	22.77±3.29	22.25±2.61
Subtype		
MO	51	-
MA	16	
CM	8	
Duration (years)	11.88±6.46	-
Frequency(days/month)	5.30±4.97	-
NRS	7.59±1.48	-
Positive family history	38(50.67%)	-
Allodynia	16(21.33%)	-
HIT-6		
Level 2	5(6.67%)	-
Level 3	7(9.33%)	
Level 4	63(84%)	

*BMI* body mass index, *NRS* numeric rating scalewith 0 indicating no pain and 10 worst possible pain, *HIT-6* headache impact test-6

### Behavioral results

**Fig. 2:**
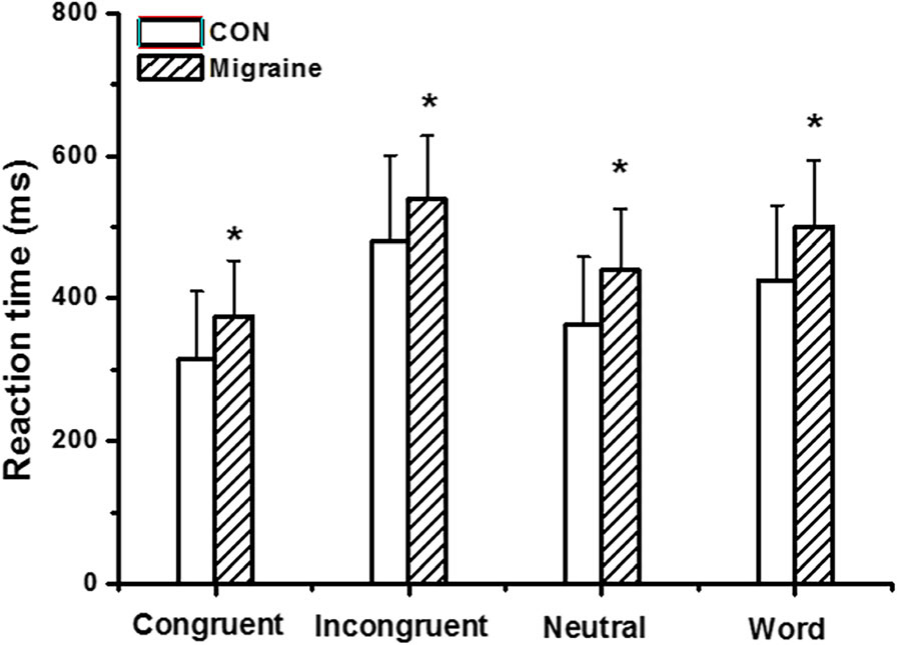
*The reaction time during different conditions.* Both the migraine and control group showed the longest reaction time linked to incongruent tasks and the shortest to congruent tasks, and the migraine group had a longer total reaction time compared to the control group under each condition

As shown in Table 2, the migraine group had a longer total reaction time compared to the control group (459.18 ± 78.13 ms versus 391.48 ± 95.21 ms, *t* = −4.12, *p* < 0.001), but there was no difference in accuracy or Stroop effect between the two groups. Repeated-measures ANOVA found a significant main effect of task type (F_(3,112)_ = 323.410, *p* < 0.001, η^2^_p_ = 0.74), with the longest reaction time linked to incongruent tasks and the shortest to congruent tasks (Fig. 2). In the subgroup analysis, the main effect of age was significant, with a greater Stroop effect among subjects aged > 30 years than in those aged < 30 years (173.78 ± 58.22 ms versus 151.97 ± 50.39 ms, *p* = 0.019). Furthermore, the interaction between group and education level was significant, with healthy subjects with more years of education (> 12 years) exhibiting a stronger Stroop effect compared to those with less education (153.97 ± 38.31 ms versus 211.90 ± 59.55 ms, *p* = 0.008); no such effect was found among migraine patients. Moreover, no obvious main effect of migraine subtype, headache duration, headache frequency, or allodynia was found in the analysis of the Stroop data.

**Table 2:** Behavioral data

	Control(*n* = 41)		Migraine(*n* = 75)	
	Mean	SD	Mean	SD
RT (ms)	391.48	95.21	459.18*	78.13
Accuracy (%)	92.20	4.34	89.87	6.57
Stroop effect (ms)	164.57	59.28	163.70	54.04

*p<0.05 compared to control group

### ERP data

#### MFN

**Fig. 3:**
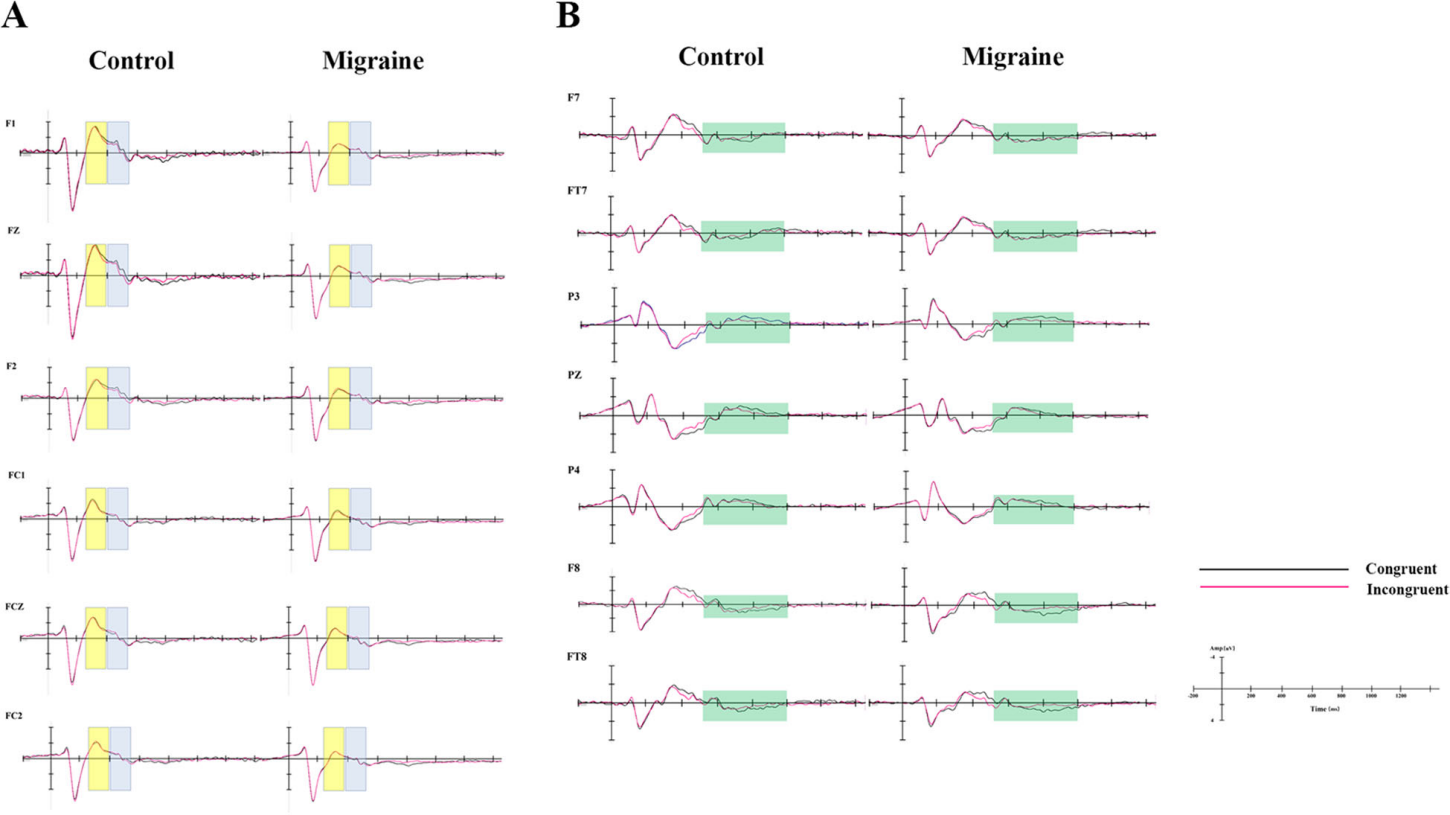
*The grand averaged ERPs elicited by task stimuli in patients and controls, respectively.* **a** Grand average ERP waveforms recorded at six frontal midline electrodes (F1, FZ, F2, FC1, FCZ, FC2) evoked by congruent tasks (black lines) and incongruent tasks (red lines), with the yellow and blue boxes referred to the early MFN and late MFN respectively. **b** Grand average ERP waveforms recorded at three regions (left frontal, right frontal and parietal regions) evoked by congruent tasks (black lines) and incongruent tasks (red lines), with the SP component marked by green boxes

With regard to the amplitude of the early MFN component (Table 3), the main effect of group was marginally significant (F_(1,114)_ = 20.81, *p* < 0.001, η^2^_p_ = 0.15), with a lower early negative MFN amplitude in the migraine group compared to the control group (Fig. 3a). The main effect of task type was not significant, but the interaction between task type and electrode site was, with a lower negative amplitude found for incongruent tasks compared to congruent tasks at the FC1 site (*p* = 0.009). In the subgroup analysis, the interaction between allodynia and electrode site was significant, with patients exhibiting allodynia having a lower negative early MFN amplitude than patients without allodynia. No other significant main effects or interactions were found.

**Table 3:** MFN mean amplitudes during the congruent and incongruent conditions at six midline frontal electrodes (F1, FZ, F2, FC1, FCZ, FC2)

	Early MFN				Late MFN			
	Congruent		Incongruent		Congruent		Incongruent	
	Control	Migraine	Control	Migraine	Control	Migraine	Control	Migraine
F1	-3.05(1.34)	-1.95(1.59)	-3.02(1.17)	-2.04(1.51)	-1.99(0.98)	-1.46(1.45)	-1.69(0.92)	-1.45(1.34)
FZ	-3.39(1.57)	-1.98(1.66)	-3.37(1.45)	-2.09(1.60)	-2.27(1.01)	-1.48(1.43)	-2.03(0.94)	-1.44(1.43)
F2	-3.16(1.53)	-1.83(1.58)	-3.16(1.37)	-2.01(1.49)	-2.33(1.02)	-1.48(1.39)	-2.02(0.96)	-1.42(1.29)
FC1	-3.06(1.41)	-1.94(1.36)	-2.84(1.36)	-1.81(1.36)	-1.34(0.79)	-0.95(1.11)	-1.38(0.69)	-1.04(1.15)
FCZ	-3.32(1.56)	-2.01(1.59)	-3.22(1.51)	-1.97(1.57)	-1.59(0.95)	-1.03(1.28)	-1.74(0.84)	-1.12(1.26)
FC2	-2.85(1.48)	-2.07(1.56)	-2.90(1.43)	-1.67(1.41)	-1.58(1.01)	-0.97(1.12)	-1.58(0.94)	-1.02(1.16)

With regard to the amplitude of the late MFN component (Table 3), the main effect of group was also significant (F_(1,114)_ = 7.65, *p* = 0.007, η^2^_p_ = 0.063), with the migraine group exhibiting a lower negative late MFN amplitude than the control group under both task types (Fig. 3a). The interaction between task type and electrode site was significant, with a lower negative amplitude for incongruent tasks relative to congruent tasks at the F2 site (*p* = 0.029). In the analysis of the effects of headache characteristics on MFN, the main effect of headache type was significant, with a more negative late MFN amplitude in MA patients compared to MO and CM patients, whereas no significant difference was found between the latter two groups (F_(2,72)_ = 3.14, *p* = 0.049, η^2^_p_ = 0.08). No other main effect or interaction was significant.

#### SP

With regard to the amplitude of the SP component (Table 4), the main effect of task type was significant in both the right lateral frontal (F_(1,114)_ = 7.63, *p* = 0.007, η^2^_p_ = 0.063) and parietal areas (F_(1,114)_ = 25.88, *p* < 0.001, η^2^_p_ = 0.19), with a lower positive SP amplitude recorded during incongruent tasks than during congruent tasks (Fig. 3b). However, no main effect of group was found; i.e., there was no significant difference between the migraine and control groups. In the analysis of the effects of headache characteristics on SP, no main effect of migraine subtype, headache duration, headache frequency, or allodynia was found. Of note, the interaction between allodynia and electrode site was significant, with a lower positive amplitude at the F8 site in migraine patients with allodynia than in patients without (*p* = 0.018).

**Table 4:** SP mean amplitudes during the congruent and incongruent conditions at three regions

		Congruent		Incongruent	
		Control	Migraine	Control	Migraine
Left frontal region	F7	2.72(0.89)	2.29(1.39)	2.45(1.07)	2.23(1.29)
	FT7	2.09(0.97)	2.16(1.27)	1.97(0.83)	2.02(1.13)
Right frontal region	F8	2.83(1.07)	2.48(1.24)	2.43(0.96)	2.31(1.51)
	FT8	2.32(0.88)	2.25(0.94)	1.91(0.67)	2.05(1.30)
Parietal region	P3	1.51(0.69)	1.60(1.01)	1.32(0.63)	1.36(0.98)
	PZ	2.05(0.87)	2.28(1.14)	1.78(0.82)	1.95(1.13)
	P4	1.63(0.85)	1.55(1.09)	1.35(0.71)	1.30(1.05)

## Discussion

In this study, we used the classic color–word Stroop paradigm, which have been widely used to examine the ERP correlates of cognitive processes related to selective attention [17, 20]. To date, no consistent or positive behavioral results have been obtained in studies investigating the attention function in migraine using the Stroop task or related paradigms [2, 10, 21, 22]. Hence, we tried to elucidate this cognitive process in migraineurs in greater detail using the ERP method. As shown in previous studies, age and education are essential factors in cognitive assessment; hence, 75 migraine patients and 41 age-, gender-, and education-matched healthy controls were enrolled in this study. Furthermore, patients with anxiety or depression were excluded, as mood disorders also significantly affect cognitive processes [3]. Thus, in this study, we aimed to determine whether migraine per se changed patients’ inhibitory control functions.

Women comprised a larger proportion (68 %) of all recruited patients, consistent with previous reports. Among our patients, 48 % reported positive family histories of migraine, lower than the rate reported in previous research (60 %) [1]. As for headache severity, more than 80 % of patients were classified as level 4 in the HIT-6 assessment, probably the main reason for their seeking medical advice. Of note, in this study, a specific clinical sign, allodynia, was assessed, and 21.33 % of all included patients reported this phenomenon, lower than in previous studies (60–80 %) [23, 24]. Consistent with previous studies, more chronic migraine patients reported allodynia than episodic ones (25 % vs. 21 %) in our cohorts. Generally, allodynia is recognized as a sign of pain chronicity resulting from the pathophysiological process of sensitization [25]. In this study, we focused on this clinical symptom to further explore cognitive alterations and migraine chronicity.

As for behavioral results, migraine patients had significantly longer reaction times than the control group, although accuracy was similar, indicating decreased executive function among migraine patients. A significant Stroop effect was observed in both groups, with longer reaction times in incongruent trials than in congruent ones. However, the two groups exhibited no significant difference in terms of scores on the Stroop test. The increase in Stroop effect is thought to result from a decline in the efficiency of inhibitory processes, and we found no decline in attentional inhibition among migraine patients at the behavioral level. A significant main effect of age on the Stroop score was noted, consistent with previous studies, which have shown decreased conflict control with aging [13]. Interestingly, we found a weaker Stroop effect among healthy controls with lower education levels than among those with more education, although no such effect of education was found among migraine patients. Generally, education has a positive influence on cognitive abilities, and why it does not in migraineurs remains to be investigated. One hypothesis is that migraine may have an impact on inhibitory processes, which are stronger among patients with higher education levels.

Focusing on the electrophysiological data, migraine patients exhibited altered inhibitory processes, as they had lower negative MFN amplitudes. This study replicated the results of previous studies by finding that subjects had lower negative MFN amplitudes during incongruent trials compared to congruent trials [16]. MFN, also known as N450 in some studies, is mainly distributed over the midline of the scalp (extending from the frontal to the parietal region) and is generally thought to represent the cognitive process of conflict detection [15, 17]. In some studies, MFN is separated into two subcomponents, the early one representing stimulus conflict detection and the late one representing response conflict detection. The grand-averaged data in this study also revealed that MFN had two peaks, at 250 and 550 ms after stimulus onset, and was divided into the early and late components at 400 ms. In this study, migraine patients exhibited less negative early MFN and late MFN, independent of confounding factors such as age and education, indicating decreased ability in both stimulus and response conflict detection. In previous studies, MFN has been associated with neural activity in the anterior cingulate cortex (ACC) or medial frontal cortex [17]. Furthermore, the disrupted functional connectivity of the central executive network centered in both ACC and middle frontal gyrus (MFG) has been demonstrated in patients with migraine with and without aura [26, 27].Therefore, we can reasonably speculate that a decline in conflict detection on the part of migraine patients may result from functional changes in these brain regions. Of note, we also found a significant main effect of migraine subtype in the subgroup analysis, with MA associated with a negative late MFN amplitude compared with MO and CM, independent of headache duration, headache frequency, or other factors, illustrating the point that patients with MA experience a less severe decline in response conflict detection than do patients with MO or CM. The underlying significance of this finding needs to be further explored. One possibility is that the alteration in brain function is relatively more localized in MA, as CM mostly develops from MO.

The SP, a sustained centroparietal positivity that follows the MFN beginning at approximately 500 ms after stimulus onset, is sensitive to conflict adaptation effects[17]. According to previous studies, SP is more positive in incongruent than in congruent trials, likely reflecting a signal for increased recruitment of cognitive control resources for accurate task completion. However, in this study, no significant difference in SP amplitude was found between migraine patients and healthy controls, indicating normal conflict control processing among patients with migraine. Group differences were not significant in terms of behavioral results, although the migraine group exhibited a significant decline in conflict detection according to neuropsychological measures of MFN. How the migraine patients managed to preserve task performance in this situation is an interesting question. One interpretation is that conflict resolution ability is relatively preserved among migraine patients, as the migraine group did not exhibit changes in SP. Therefore, the slower processing at the early conflict detection stage neither slackened the subsequent conflict resolution processing stage for compensatory adjustments for accurate task completion nor affected the behavioral results. Although the group differences in SP amplitude and the behavioral results were not significant, behavioral effects may be more obvious with a more difficult task. From another perspective, the non-significant effect at the behavioral level might also have been due to the low sensitivity of the behavioral measure used.

In this study, we focused on a specific clinical sign of migraine, allodynia. Allodynia has been long recognized as a clinical symptom of sensitization in chronic pain as well as an independent predictor of migraine chronification [28]. Accordingly, 60–80 % of migraine patients report experiencing allodynia, with increased sensitivity to normally non-painful stimulation of the head and even the whole body, such as by touching, combing, and so on [23]. In our cohort, only 21.18 % reported allodynia during the ictal or interictal period, which might have resulted from the low proportion of chronic migraine patients, as allodynia is more frequent among chronic migraine patients [28]. Interestingly, some positive results were obtained in subgroup analysis using allodynia as a within-subject factor. On the one hand, migraine patients who experienced allodynia had lower negative early MFN relative to those without allodynia at F1; on the other hand, patients with allodynia exhibited less positive SP at F8. These data suggest that the migraine sufferers with allodynia had, to some extent, experienced a decline in both conflict detection and conflict resolution, supporting the argument that migraine patients exhibit decreased inhibitory control in the process of chronification. Indeed, allodynia has been associated with increased cognitive networks disarrangement in recent neuroimaging studies, as significantly reduced functional connectivity of both default mode network and central executive network has been observed among migraine patients with cutaneous allodynia than those without [29]. Especially, the reduced functional connectivity of ACC and MFG may represent the neuronal substrate of both subclinical impairment of complex executive functions and dysfunction of antinociceptive pathway, making migraine patients more inclined to chronic migraine. However, no obvious difference between chronic migraine and episodic migraine was found in subgroup analysis, which seems contradictory to the above speculation. This might have been due to the unbalanced sample sizes among types of migraine. An enlarged sample size should provide more forceful evidence.

Thus, we revealed changes in the inhibitory process among migraine patients during the interictal phase, supporting another aspect of cognitive decline in migraine. This decline may correspond to functional alterations in specific brain areas. Migraine is now widely recognized as a disorder in brain function. Numerous functional studies have pointed to alterations in related brain regions, mainly including the cortex, limbic system, and brainstem [30]. Of these, the prefrontal lobe and ACC may be responsible for the cognitive and emotional aspects of migraine. The electrophysiological abnormalities detected in this study probably also resulted from dysfunction in these regions, as previous source localization demonstrated. Even though no obvious attentional decline in behavioral level, the significant electrophysiological changes in this study may underlie a subclinical difficulty affecting the executive functions making migraine patients more prone to the development of migraine attacks when exposed to daily living difficulties, as these disturbances have been recognized as common triggers for migraine attacks. From this, the cognitive decline probably in turn promotes the chronification process of migraine. Therefore, our findings, to some extent, indicate the significance of cognitive behavioral therapy for this disorder [31, 32], not only relieving cognitive disturbances but also preventing migraine chronification.

Above all, this study evaluated the attentional inhibition function in interictal migraine with both behavioral and neuropsychological measures using the Stroop task. Several meaningful points were confirmed in this paper. Furthermore, there is much more work to be done in the future. First, our small cohort of chronic migraineurs limited the examination of differences between chronic migraine and episodic migraine, so the inhibitory function assessment of chronic migraine needs to be expanded. Second, to eliminate the influence of possible compounding factors, patients with medication use, anxiety, and/or depression were all excluded from this study; however, these are common conditions in migraine, and these compounding factors should be further analyzed. Third, we focused on limited previously demonstrated components at some specific electrode sites, and examination of multi-electrode sites with possible source analysis or a combination of functional neuroimaging methods may provide more details in the future. Last but not least, accumulating evidence has revealed cognitive changes associated with migraine, but the interaction between the two and the underlying pathophysiology remain to be investigated.

## Conclusions

In conclusion, in this study, declines in attentional inhibition functions at the electrophysiological level were found among migraine patients during the interictal phase, independent of compounding factors such as age and education. Such declines mainly focused on the conflict-monitoring stage, as migraine patients displayed an obvious difference in MFN amplitude compared to healthy controls. Of note, migraine patients with allodynia exhibited some significant differences in early MFN and SP compared to those without, emphasizing the potential of considering an aggravation in decline in inhibitory control due to migraine chronicity. Our findings also have important implications for further studies of attentional evaluation in migraine and thus provide empirical support for clinical behavioral therapy.

## Data Availability

The data of the present study are available from the corresponding authors on reasonable request.
